# Urinary phosphate-containing nanoparticle contributes to inflammation and kidney injury in a salt-sensitive hypertension rat model

**DOI:** 10.1038/s42003-020-01298-1

**Published:** 2020-10-15

**Authors:** Qin Wang, Kenichi Ishizawa, Jinping Li, Wataru Fujii, Yoshikazu Nemoto, Osamu Yamazaki, Yoshifuru Tamura, Yutaka Miura, Xuedan Nie, Ryo Abe, Hiroko Segawa, Makoto Kuro-O, Shigeru Shibata

**Affiliations:** 1 Division of Nephrology, Department of Internal Medicine, Teikyo University School of Medicine, Tokyo, 173-8605 Japan; 2 Department of Nephrology, The Second Affiliated Hospital of Harbin Medical University, Harbin, 150081 China; 3 Department of Nephrology, Tianjin First Central Hospital, Tianjin, 300000 China; 4 Division of Anti-aging Medicine, Center for Molecular Medicine, Jichi Medical University, Tochigi, 329-0498 Japan; 5 Department of Neurology, The Second Affiliated Hospital of Harbin Medical University, Harbin, 150081 China; 6 Strategic Innovation and Research Center, Teikyo University, Tokyo, 173-8605 Japan; 7 Department of Applied Nutrition, Institute of Biomedical Sciences, Tokushima University Graduate School, Tokushima, 770-8503 Japan

**Keywords:** Kidney diseases, Kidney, Inflammation

## Abstract

Although disturbed phosphate metabolism frequently accompanies chronic kidney disease (CKD), its causal role in CKD progression remains unclear. It is also not fully understood how excess salt induces organ damage. We here show that urinary phosphate-containing nanoparticles promote kidney injury in salt-sensitive hypertension. In Dahl salt-sensitive rats, salt loading resulted in a significant increase in urinary phosphate excretion without altering serum phosphate levels. An intestinal phosphate binder sucroferric oxyhydroxide attenuated renal inflammation and proteinuria in this model, along with the suppression of phosphaturia. Using cultured proximal tubule cells, we confirmed direct pathogenic roles of phosphate-containing nanoparticles in renal tubules. Finally, transcriptome analysis revealed a potential role of complement C1q in renal inflammation associated with altered phosphate metabolism. These data demonstrate that increased phosphate excretion promotes renal inflammation in salt-sensitive hypertension and suggest a role of disturbed phosphate metabolism in the pathophysiology of hypertensive kidney disease and high salt-induced kidney injury.

## Abstract (web summary)

Qin Wang et al. show that altered phosphate metabolism contributes to the progression of kidney disease using a salt-sensitive rat model. They demonstrate that using the phosphate binder sucroferric oxyhydroxide reduces kidney injury and, using transcriptome sequencing, identify a potential role for complement C1q in kidney inflammation linked to phosphate metabolism defects.

## Introduction

Altered phosphate metabolism is frequently associated with chronic kidney disease (CKD)^1–3^. Because phosphate is mainly excreted from the kidney, the decreased kidney function results in positive phosphate balance in the body, which triggers the secretion of a phosphaturic hormone fibroblast growth factor 23 (FGF-23) in osteocytes. FGF-23 then acts on renal proximal tubules by binding to a complex of αKlotho and FGF receptor, increasing fractional excretion of phosphate (FE_P_) and preventing the elevation in serum phosphate levels^1^. While this process is considered to be an appropriate physiological adaptation, epidemiological studies found that it is also associated with the progression of CKD^4–7^. In these studies, the elevation in serum FGF-23, an early indicator of disturbed phosphate metabolism in CKD, has been shown to be independently associated with greater risk of end-stage renal disease (ESRD)^4–7^. However, little is known about the molecular basis that underlies the association.

Hypertension continues to be a global health problem worldwide, being a principal risk factor for cardiovascular and kidney diseases^8–10^. Although the precise mechanism of hypertension remains elusive owing to the trait’s complexity, studies to date have established the central role of the kidney and dietary salt (NaCl)^11–13^. Moreover, in addition to the development of hypertension, several lines of evidence indicate that excessive salt intake directly promotes cardiovascular and renal damage particularly in salt-sensitive hypertension^14–16^. Prospective studies in essential hypertension demonstrated that the cardiovascular events occurred more frequently in patients who had salt-sensitive hypertension than those who were not salt-sensitive^14^. A close relationship between salt sensitivity and cardio-renal damage was also demonstrated in other studies using different cohorts^15,16^. Although these data indicate an additional role of excess salt in promoting target organ damage independently of blood pressure elevation, the underlying mechanisms are not fully elucidated.

Despite the high prevalence of hypertension as well as the widespread use of phosphate as a food preservative^17^, the contribution of disturbed phosphate metabolism in hypertensive kidney disease remains unknown. In this study, we here tested the role of phosphate in the pathogenesis of kidney injury associated with salt-sensitive hypertension.

## Results

### Intestinal phosphate binding ameliorates glomerulosclerosis and tubulointerstitial damages in Dahl salt-sensitive (Dahl/SS) rats

**Fig. 1:**
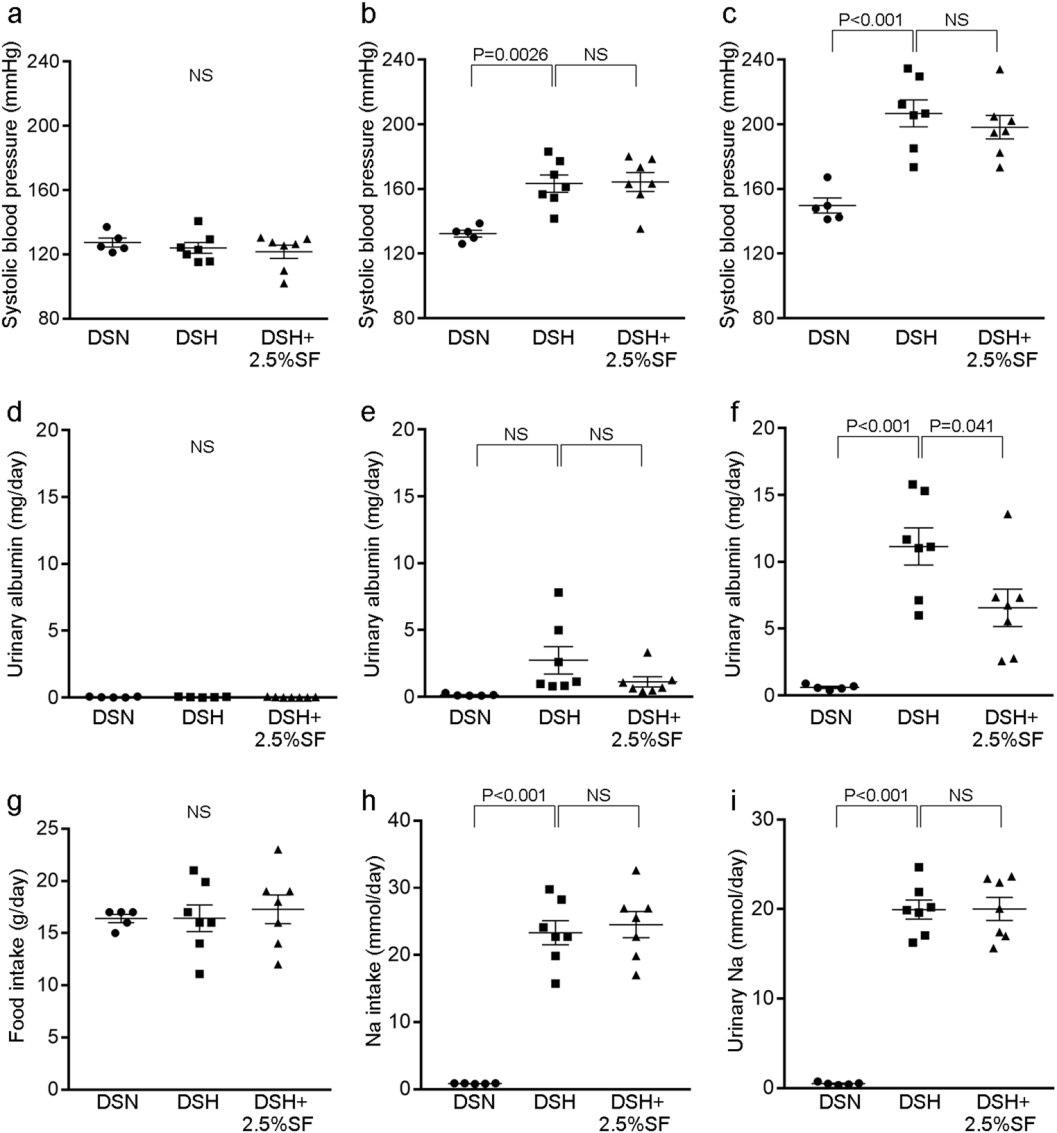
Albuminuria in Dahl salt-sensitive hypertensive rats is ameliorated by inhibiting intestinal phosphate absorption. **a**–**f** Systolic blood pressure (**a**–**c**) and urinary albumin (**d**–**f**) levels in Dahl salt-sensitive (Dahl/SS) rats receiving normal salt (DSN), high salt (DSH), and high salt with 2.5% sucroferric oxyhydroxide (DSH+2.5%SF) at 0 weeks (**a**, **d**), 2 weeks (**b**, **e**), and 4 weeks (**c**, **f**). **g**–**i** Food intake (**g**), Na^+^ intake (**h**), and urinary Na^+^ levels (**i**) at 4 weeks in DSN, DSH, and DSH+2.5%SF rats. Data are expressed as mean ± SEM; *n* = 5 or 7 animals per group; NS not significant.

Male Dahl/SS rats were randomly assigned to the control, normal-salt diet (DSN), the high-salt diet (DSH), and the high-salt diet with sucroferric oxyhydroxide (SF; 25 mg/g chow; DSH+2.5%SF) groups. Phosphorus levels in the diet were kept normal (0.3%; AIN-93G) in all the groups. Compared with DSN, DSH rats showed a significant increase in systolic blood pressure at 2 and 4 weeks (Fig. 1a–c). Blood pressure levels in the DSH+2.5%SF group were identical to those in the DSH groups throughout the experimental period (Fig. 1a–c). DSH rats had a progressive increase in urinary albumin levels (Fig. 1d–f) and showed marked albuminuria compared with the DSN group at 4 weeks (DSH, 11.15 ± 1.40 mg/day versus DSN, 0.61 ± 0.08 mg/day; *P* < 0.001; Fig. 1f). Interestingly, however, we found that DSH+2.5%SF rats showed significantly lower levels of albuminuria than DSH rats (6.56 ± 1.39 mg/day; *P* = 0.041; Fig. 1f). This difference may not be attributable to the hemodynamic changes, because blood pressure levels were identical between the DSH and DSH+2.5%SF groups. Moreover, food intake, Na^+^ intake, and urinary Na^+^ levels were all similar between the DSH and DSH+2.5%SF groups (Fig. 1g–i).

**Fig. 2:**
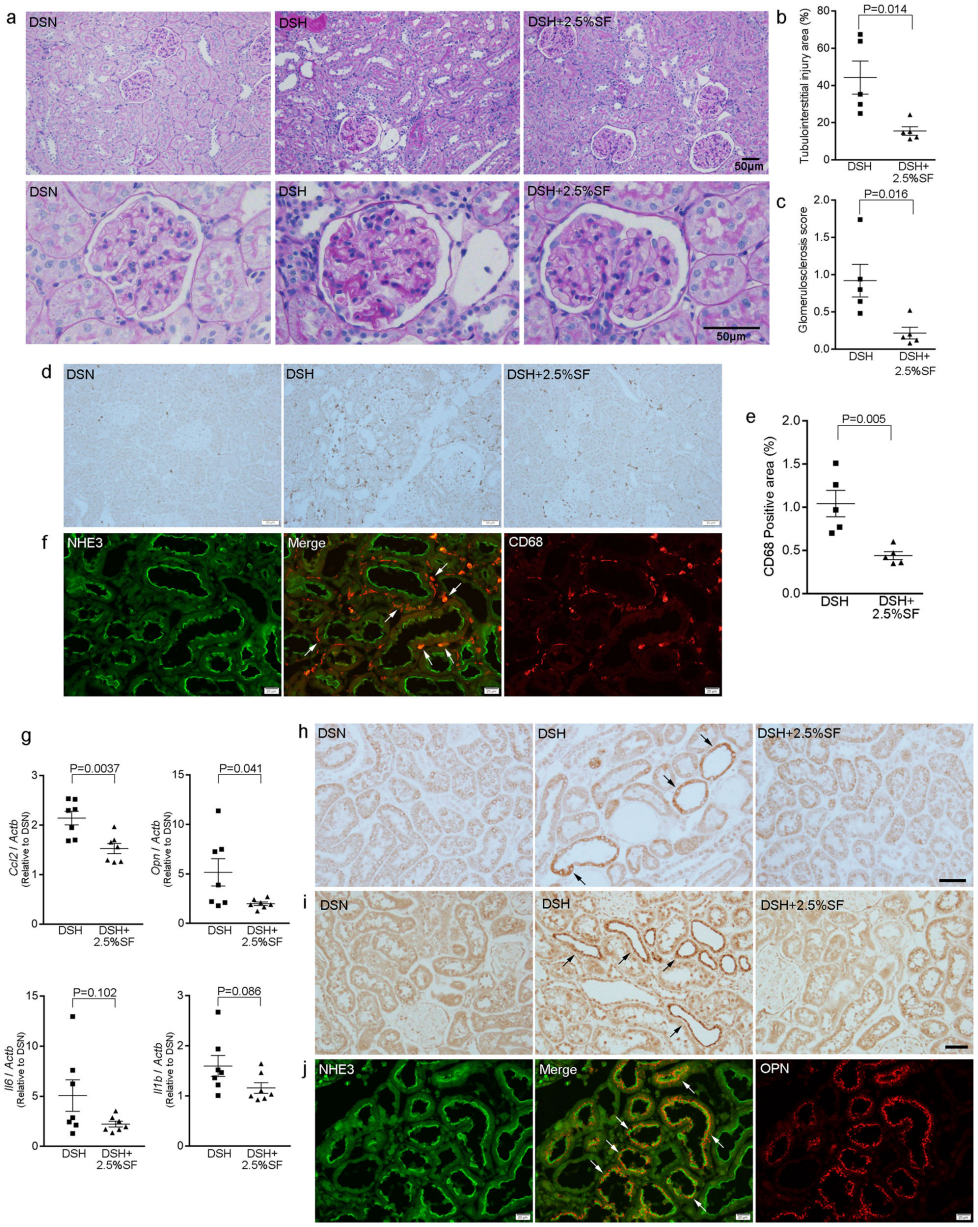
Renoprotective effects of SF are associated with the alleviation of renal inflammation. **a** Representative Periodic acid-Schiff (PAS)-stained kidney sections from DSN, DSH, and DSH+2.5%SF rats. **b**, **c** Dot plots for histological analysis of tubulointerstitial injury and of glomerulosclerosis in the indicated groups (*n* = 5 animals per group). **d** Immunohistochemical staining for ED-1 (CD68) in the kidneys of DSN, DSH, and DSH+2.5%SF rats. **e** Quantitative analysis of the CD68-positive area in the kidney (*n* = 5 animals per group). Infiltration of the CD68-positive macrophages in the kidneys of DSH rats was significantly ameliorated by inhibiting intestinal phosphate absorption using SF. **f** Kidney section from DSH rats was stained for sodium hydrogen exchanger 3 (NHE3; a marker for proximal tubules) (green) and CD68 (red). CD68-positive macrophages were present around proximal tubule cells (arrows). **g** Quantitative analysis of *Ccl2*, *Opn*, *Il6*, and *Il1b* gene expression by real time RT-PCR in the kidney (*n* = 7 animals per group). Expression levels were normalized to those of *Actb* (encoding β-actin), shown relative to DSN. **h**, **i** Immunohistochemical staining for Ccl2 (MCP-1, encoded by *Ccl2*) (**h**) and osteopontin (encoded by *Opn*) (**i**) in the kidneys of DSN, DSH, and DSH+2.5%SF rats. **j** Kidney section from DSH rats was stained for NHE3 (green) and osteopontin (OPN, red). The cells expressing osteopontin (arrows) were positive for NHE3, demonstrating that they are proximal tubule cells. Data are expressed as mean ± SEM; Bars: 20 μm in **f**, **j**; 50 μm in **a**, **d**, **h**, **i**.

We next evaluated renal histology. In the periodic acid-Schiff (PAS)-stained kidney sections, DSH rats showed glomerulosclerosis and tubulointerstitial changes including tubular dilatation, tubular atrophy, and intraluminal cast without evidence of overt calcification, a finding consistent with previous observations^18^ (Fig. 2a). Semiquantitative evaluation of renal histology demonstrated that the tubulointerstitial and glomerular changes in DSH rats were significantly alleviated by the administration of SF (Fig. 2b, c). Existing evidence suggests a role of glomerular podocyte in the development of glomerulosclerosis and the progression of renal dysfunction in CKD^19,20^. Immunohistochemical study revealed that the expression of desmin, a sensitive marker for podocyte injury^21,22^, was significantly reduced in podocytes in the glomeruli of DSH+2.5%SF rats as compared with DSH rats (Supplementary Fig. 1).

### SF alleviates renal inflammation in Dahl/SS rats

Macrophage infiltration and renal inflammation are the initial responses to the kidney injury, leading to the progression of CKD^23–25^. As shown in Fig. 2d, DSH rats showed macrophage infiltration in the tubulointerstitium, predominantly in the cortex, as assessed by immunohistochemical staining of ED-1 (CD68). Quantitative evaluation of the CD68-positive areas revealed that the positive areas in DSH+2.5%SF rats were 58% less than those in DSH rats (Fig. 2e). We used immunofluorescence (IF) microcopy to determine the nephron segments in which macrophages were present. Kidneys from DSH rats were stained with anti-CD68 and anti-sodium hydrogen exchanger 3 (NHE3), a marker of proximal tubules. The results demonstrated that CD68-positive macrophages were observed around proximal tubules in the cortex (Fig. 2f).

Renal proximal tubules are known to play a key role in the initiation and promotion of tubulointerstitial inflammation by interacting with infiltrating macrophages^26^. Previous studies have shown that proximal tubule cells can produce proinflammatory factors, such as Ccl2 (also called monocyte chemotactic protein-1 (MCP-1)) and osteopontin^27,28^, in response to various stimuli, promoting macrophage migration and tubulointerstitial inflammation. In quantitative real-time reverse transcriptase polymerase chain reaction (RT-PCR) analysis, we found that the upregulation of *Ccl2* (encoding C-C chemokine motif ligand 2 (Ccl2)) and *Opn* (encoding osteopontin) in DSH rats was significantly ameliorated in DSH+2.5%SF rats (29% decrease for *Ccl2*; *P* = 0.0037, and 62% decrease for *Opn*, *P* = 0.041; Fig. 2g). In addition, we found that the expression of macrophage-derived cytokines *Il1b* (encoding interleukin 1β (IL1β)) and *Il6* (encoding IL6) were nonsignificantly attenuated by the administration of SF (Fig. 2g). Reduced expression of *Ccl2* and *Opn* in the kidney of DSH+2.5%SF rats can result from either decreased production in renal tubules or attenuation of macrophage infiltration. To demonstrate that Ccl2 and osteopontin were reduced in renal tubules, we performed immunohistochemical analysis. The results demonstrated that the positive staining for Ccl2 in renal tubules was occasionally seen in DSH rats, whereas no such signal was observed in DSN or DSH+2.5% rats (Fig. 2h). Similarly, osteopontin was increased in renal tubules in the DSH group, which was attenuated in the DSH+2.5%SF group (Fig. 2i). These data were further confirmed by semiquantitative evaluation (osteopontin-positive area, 1.6 ± 0.2% in DSH versus 0.3 ± 0.1% in DSH+2.5%SF; *P* = 0.0011). Double IF study demonstrated that the proinflammatory factor is increased in proximal tubule cells in DSH rats (Fig. 2j). These data suggest that increased production of proinflammatory factors in renal tubules facilitates macrophage infiltration and renal inflammation in DSH rats. Moreover, the attenuation by SF suggests that these processes are in part mediated by the altered phosphate metabolism.

### Dahl/SS rats show normal serum phosphate levels but increased phosphate excretion

**Fig. 3:**
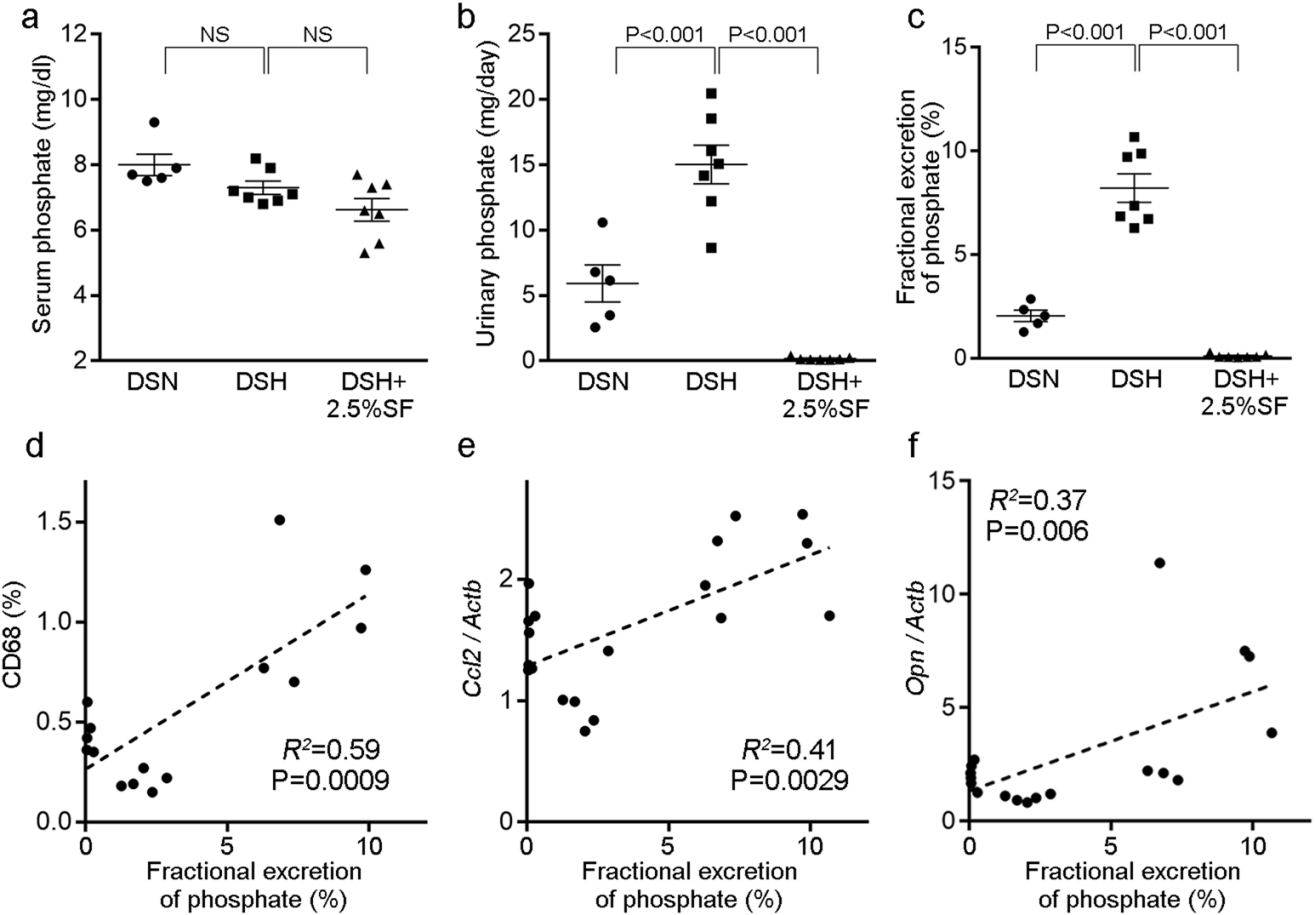
Renal inflammatory markers correlate with fractional excretion of phosphate in Dahl/SS rats. **a**–**c** Serum phosphate levels (**a**), urinary phosphate levels (**b**), and fractional excretion of phosphate (FE_P_) (**c**) in the indicated groups. Data are expressed as mean ± SEM; *n* = 5 or 7 animals per group. NS not significant. **d** Correlation between FE_P_ and CD68-positive areas in the kidney (*n* = 15 animals). **e**, **f** Correlation between FE_P_ and *Ccl2* (**e**) or *Opn* (**f**) expression in the kidney (*n* = 19 animals).

We then evaluated phosphate metabolism in Dahl/SS rats. As shown in Fig. 3a, serum phosphate levels in the DSH group were similar to those in the DSN group. Also, there was no significant difference in serum phosphate levels between DSH and DSH+2.5%SF rats. However, urinary phosphate excretion (Fig. 3b) and FE_P_ (Fig. 3c) at 4 weeks were significantly elevated in the DSH group (urinary phosphate levels, 15.03 ± 1.48 mg/day in DSH versus 5.93 ± 1.41 mg/day in DSN, *P* < 0.001; FE_P_, 8.21 ± 0.68% in DSH versus 2.05 ± 0.27% in DSN, *P* < 0.001). These changes were suppressed by the administration of SF (urinary phosphate levels, 0.17 ± 0.04 mg/day in DSH+2.5%SF, *P* < 0.001 versus DSH group; FE_P_, 0.12 ± 0.03% in DSH+2.5%SF, *P* < 0.001 versus DSH group). Thus, although DSH rats showed normal serum phosphate levels, they likely had abnormal phosphate metabolism, which was corrected by SF. Correlation analysis revealed that both CD68-positive areas (Fig. 3d) and *Ccl2* (Fig. 3e) levels were highly correlated with FE_P_. *Opn* levels were also correlated with FE_P_, although the *R*^2^ value was slightly lower (Fig. 3f). These data are consistent with the notion that the increased phosphate excretion contributes to renal inflammation in Dahl/SS rats.

In the plasma, mineral-binding proteins such as fetuin-A binds and sequesters a CaPi nanoparticle, forming a complex (calciprotein particle (CPP)). We additionally evaluated plasma CPP levels^29^. We found that CPP levels were not significantly different among three groups at this stage (Supplementary Fig. 2a). Previous studies demonstrated that plasma CPP correlates with serum phosphate and with calcium–phosphate product^30^. Consistently, we found significant correlation between CPP levels and phosphate levels (*R*^2^ = 0.39; *P* = 0.004) and also between CPP levels and calcium–phosphate product (*R*^2^ = 0.49; *P* = 0.0009) (Supplementary Fig. 2b, c).

**Fig. 4:**
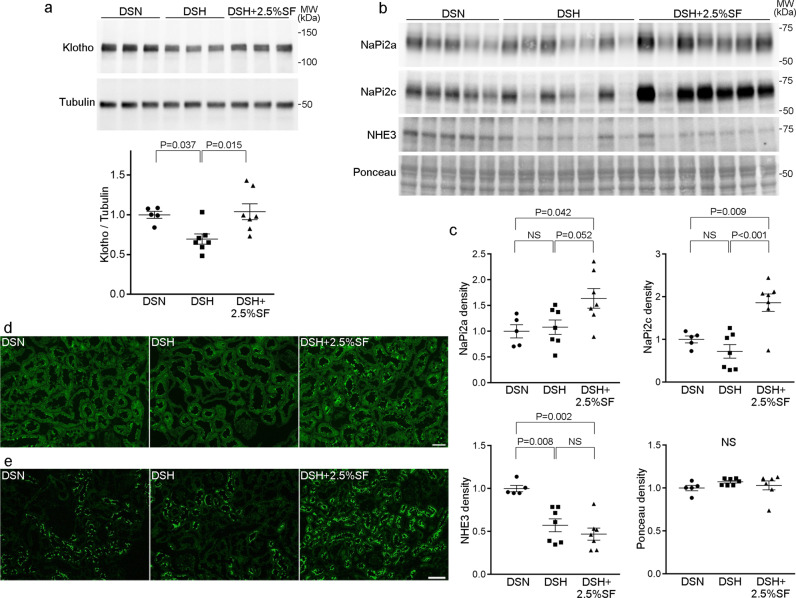
Renal Klotho, NaPi2a, and NaPi2C levels in Dahl/SS rats. **a** Renal Klotho and tubulin levels in DSN, DSH, and DSH+2.5%SF rats. Dot plots show the results of quantitation. **b**, **c** Abundance of NaPi2a, NaPi2c, and NHE3 in the plasma membrane-enriched fraction of the kidney in DSN, DSH, and DSH+2.5%SF rats. Dot plots in **c** show the results of quantitation. **d**, **e** Kidney sections from the indicated rats were stained for NaPi2a (**d**) and NaPi2c (**e**). The apical staining of NaPi2a and NaPi2c was highly increased in DSH+2.5%SF rats. Data are expressed as mean ± SEM; *n* = 5 or 7 animals per group. Bars represent 50 μm in **d**, **e**.

To obtain insights into the changes in FE_P_, we also evaluated the levels of phosphaturic hormone FGF-23, parathyroid hormone (PTH), 1,25(OH)_2_D_3_, and Na^+^-dependent phosphate transporters. The difference in PTH and 1,25(OH)_2_D_3_ levels between the DSN and DSH groups were not statistically significant (Table 1). However, we found that FGF-23 levels were significantly increased in DSH rats, which was highly significantly suppressed in DSH+2.5%SF rats (Table 1). FGF-23 levels were significantly correlated with FE_P_ (*R*^2^ = 0.48; *P* = 0.0019), explaining the changes in FE_P_ in this model at least in part. These data indicate that, although phosphate metabolism is dysregulated in our model, serum phosphate levels were kept constant through increased renal phosphate excretion. Given the evidence that chronic kidney injury and phosphaturia can alter full-length, renal Klotho expression levels^29,31^, we evaluated Klotho abundance. We found that the renal Klotho levels were lower in the DSH group than in the DSN group and that these changes were alleviated by SF (Fig. 4a).

**Table 1:** Biological parameters at 4 weeks.

Group	DSN	DSH	DSH+2.5%SF
Ccr (ml/min/100 g)	0.85 ± 0.11	0.65 ± 0.09	0.62 ± 0.03
Body weight (g)	280 ± 5	277 ± 3	259 ± 3^a,b^
ΔBody weight (g)	178 ± 6	176 ± 6	164 ± 5
Serum Na (mmol/l)	143 ± 0.3	141 ± 0.7	141 ± 0.5
Serum Cl (mmol/l)	101 ± 0.9	98 ± 0.9	98 ± 1.4
Serum K (mmol/l)	3.8 ± 0.3	3.5 ± 0.2	3.5 ± 0.3
Serum HCO_3_ (mmol/l)	27.1 ± 1.0	28.9 ± 0.5	28.0 ± 1.0
Hemoglobin (g/dl)	13.8 ± 0.2	12.7 ± 0.9	13.8 ± 0.6
Serum Ca (mg/dl)	10.7 ± 0.2	10.8 ± 0.1	10.8 ± 0.2
Intact PTH (pg/ml)	106.8 ± 8.0	216.2 ± 69.8	44.8 ± 23.6^c^
1,25(OH)2D3 (pg/ml)	467 ± 35	373 ± 34	776 ± 86^a,b^
FGF-23 (pg/ml)	740.9 ± 60.4	1193.0 ± 147.6^d^	371.3 ± 44.1^e^

^a^*P* < 0.01 versus DSN; ^b^*P* < 0.01 versus DSH; ^c^*P* < 0.05 versus DSH; ^d^*P* < 0.05 versus DSN; ^e^*P* < 0.001 versus DSH.

NaPi2a (Npt2a, encoded by *Slc34a1*) and NaPi2c (Npt2c, encoded by *Slc34a3*) are present in the apical membrane of the proximal tubules, regulating Na^+^-dependent phosphate reabsorption in the kidney^3,32^. We next evaluated NaPi2a and NaPi2c abundance in our model. Western blot analysis revealed that both NaPi2a and NaPi2c levels were significantly increased in DSH+2.5%SF rats compared with those in DSN rats (1.6-fold increase and 1.9-fold increase, respectively) (Fig. 4b, c). NaPi2c levels in the DSH group seemed modestly decreased compared with DSN rats, although the difference is not statistically significant (Fig. 4b, c). Consistent with the results of western blotting, IF study demonstrated strong apical staining of NaPi2a and NaPi2c in the kidneys of DSH+2.5%SF rats (Fig. 4d, e). We additionally evaluated the levels of NHE3, a major Na^+^ transporter expressed in proximal tubule cells. In contrast to the findings in NaPi2a and NaPi2c, NHE3 levels in DSH+2.5% rats were significantly suppressed compared with DSN rats (Fig. 4b, c). NHE3 abundance in DSH was also suppressed, to a similar extent as that of the DSH+2.5%SF group (Fig. 4b, c). These data indicate that reduced phosphate intake in DSH+2.5%SF rats results in the upregulation of NaPi transporters in the kidney, thereby suppressing FE_P_ in this group. The changes in NHE3 abundance in DSH and DSH+2.5%SF rats are likely attributable to the increased Na^+^ intake (Fig. 1h) and compensatory decrease in renal Na^+^ reabsorption.

### SF alleviates macrophage infiltration but not hypertrophy in the heart of Dahl/SS rats

**Fig. 5:**
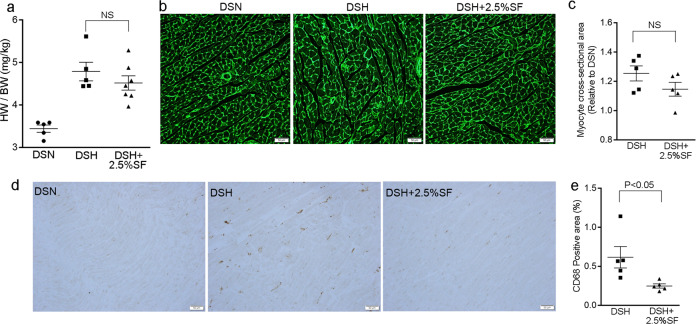
SF ameliorates heart inflammation. **a** Heart weight-to-body weight ratio (HW/BW) in DSN, DSH, and DSH+2.5%SF rats (*n* = 5 or 7 animals per group). **b** Wheat germ agglutinin (WGA) staining for heart cross-sections. **c** Quantitative analysis of the cardiomyocyte cross-sectional area (*n* = 5 animals per group). **d** Immunohistochemical staining for CD68 in the heart of DSN, DSH and DSH+2.5%SF rats. **e** Quantitative analysis of the CD68-positive area in the heart (*n* = 5 animals per group). Infiltration of the CD68-positive macrophages in the heart of DSH rats was significantly ameliorated by SF. Data are expressed as mean ± SEM; NS not significant. Bars represent 50 μm in **b**, **d**.

The elevation of FGF-23 levels is associated with left ventricular hypertrophy in CKD^3^, and several possibilities have been postulated as the underlying mechanisms^33^. Given the significant changes in FGF-23 levels among the three groups (Table 1), we next performed morphological analysis of the cardiac tissue. Consistent with the high blood pressure in the DSH group, heart weight-to-body weight (HW/BW) ratio was significantly greater in this group than in the DSN group (Fig. 5a). We observed similar increases in HW/BW ratio in the DSH+2.5%SF group (Fig. 5a). Consistently, quantitative evaluation of myofiber cross-sectional areas in wheat germ agglutinin (WGA)-stained sections indicated that the individual myofiber diameter was comparable between the DSH and DSH+2.5%SF groups (Fig. 5b, c). Of interest, however, CD68 staining of the heart revealed that macrophage infiltration in DSH was significantly reduced by SF (60% decrease; *P* = 0.03; Fig. 5d, e). Given that blood pressure levels were similarly increased in DSH and DSH+2.5%SF rats, these data indicate that high blood pressure is the major determinant of cardiac hypertrophy in Dahl/SS rats; however, they also suggest a possible contribution of abnormal phosphate metabolism in cardiac inflammation.

### Phosphate loading directly induces MCP-1 gene expression in NRK-52E cells

Previous micro-puncture study demonstrated that the phosphate concentrations in tubular fluid can increase in pathological conditions that stimulate phosphaturic hormones such as FGF-23 and PTH^34,35^. Consistently, we found that FE_P_ in DSH was four times higher than in DSN rats, which is, at least in part, explained by the changes in serum FGF-23 levels. To determine whether phosphate loading to renal tubules directly triggers inflammation, we analyzed the effects of inorganic phosphate (Pi) in cell culture.

**Fig. 6:**
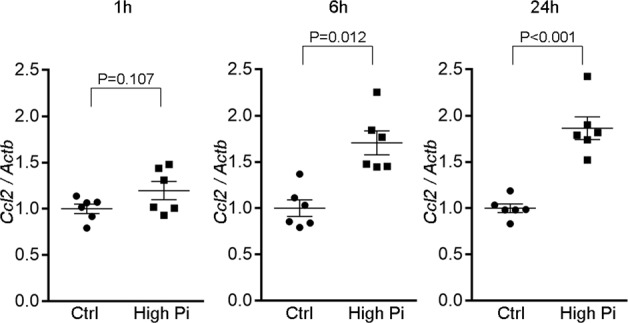
Phosphate loading directly induces *Ccl2* expression in proximal tubule cells. NRK-52E cells, a proximal tubule cell line, were incubated with high inorganic phosphate (Pi) for 1, 6, and 24 h (*n* = 6 per group). Expression levels were normalized to those of *Actb*. Ctrl control; Data are expressed as mean ± SEM.

Previous studies demonstrated that renal tubules can produce chemokine Ccl2 in response to various stimuli^36,37^; however, the effects of phosphate are not known. Using rat proximal tubule cell line, NRK-52E cells, we tested whether phosphate loading increases the expression of *Ccl2*. The cells were incubated with normal (1 mM) or high (~3 mM) Pi medium. Phosphate loading significantly increased the expression of *Ccl2* at 1, 6, and 24 h (Fig. 6). We also tested whether phosphate could induce *Opn*. However, we did not observe significant changes in *Opn* expression in our experimental condition (Supplementary Fig. 3), suggesting that several different mechanisms can act in parallel to mediate the upregulation of proinflammatory factors in renal tubules; urinary phosphate can directly induce *Ccl2* in proximal tubules, whereas *Opn* may be induced in a secondary or more complicated manner, such as the interaction between renal tubules and infiltrating macrophages^38^. In the following cell culture experiments, we focused on the regulation of *Ccl2* expression.

**Fig. 7:**
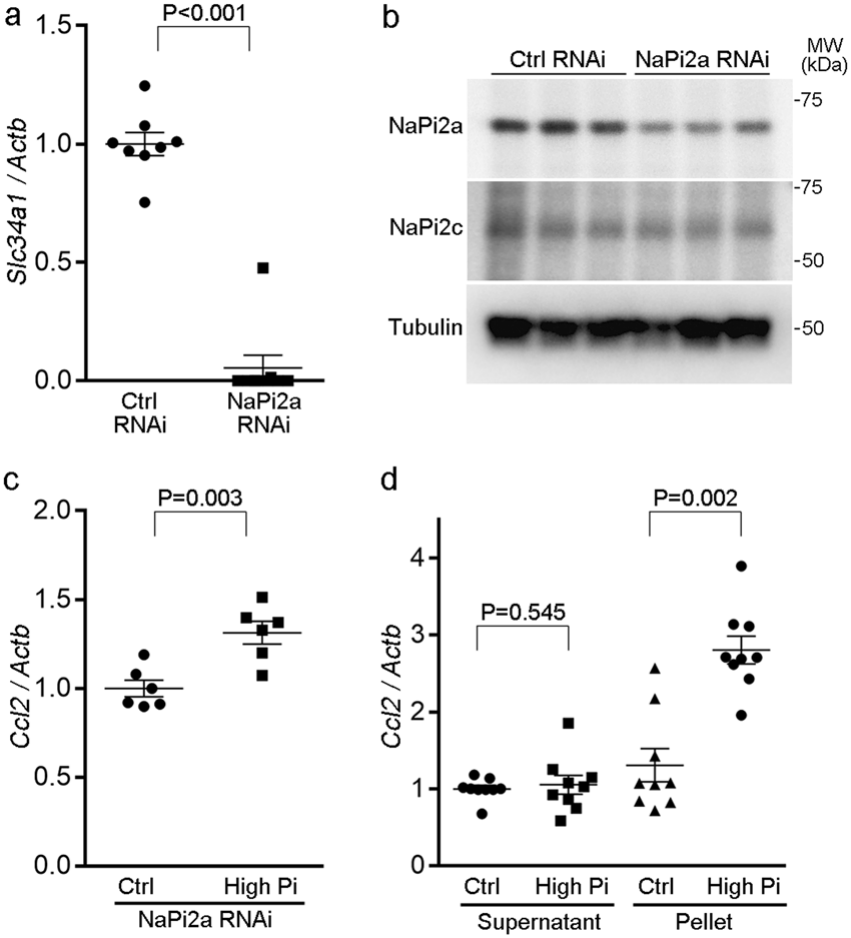
Phosphate-induced nanocrystals, rather than free Pi, enhance *Ccl2* expression in NRK-52E cells. **a**, **b** Knockdown efficiency of siRNA targeting NaPi2a (encoding *Slc34a1*) in NRK-52E cells, as analyzed by quantitative RT-PCR (**a**) (*n* = 8 or 9) and by western blot analysis (**b**). **c** Quantitative analysis of *Ccl2* expression by real-time RT-PCR in the indicated groups. NRK-52E cells were introduced with siRNA targeting NaPi2a or control siRNA. Cells were then incubated with or without Pi for 24 h (*n* = 6). **d** Effect of control or high-Pi medium supernatants or pellet resuspensions (nanocrystals) on *Ccl2* expression after 24 h (*n* = 9). Expression levels were normalized to those of *Actb*. Data are expressed as mean ± SEM.

The induction of *Ccl2* by phosphate loading in proximal tubules can physically be triggered either by Pi entry into the cells through NaPi transporters or by the formation of nanoparticles containing CaPi nanocrystals (CPPs)^1,3^. To dissect the mechanisms, we first knocked down NaPi2a (a major form of renal phosphate transporter in rodent proximal tubules^39^) by RNA interference. The introduction of small interfering RNA (siRNA) targeting NaPi2a successfully reduced the expression of *Slc34a1* (encoding NaPi2a) by 94% (Fig. 7a). Moreover, western blot analysis demonstrated the reduction in NaPi2a protein abundance (Fig. 7b). We also confirmed that the knockdown of NaPi2a did not accompany compensatory increase in NaPi2c (Fig. 7b). However, high Pi exposure for 24 h was still capable of inducing *Ccl2* in the absence of NaPi2a (Fig. 7c).

Next, we isolated CaPi nanocrystals from high-Pi medium by centrifugation^40^ and resuspended them in the control medium for comparison with the supernatant. Control or high-Pi medium was incubated at 37 °C for 3 days and then centrifuged at 16,000 × *g* for 1 h^40^. The pellets (CaPi nanocrystals), resuspended in fresh control medium (pellet resuspensions), and the supernatants were then each used to treat cells and RNA was extracted after 24 h. The high-Pi pellet resuspension significantly induced *Ccl2* expression by 2.8-fold compared with control medium, whereas the high-Pi supernatant did not (Fig. 7d). Taken together, high-Pi-induced nanocrystals, rather than free Pi, can directly induce *Ccl2* in NRK-52E cells.

### Transcriptome analysis reveals a potential role of complement in renal inflammation associated with altered phosphate metabolism in Dahl/SS rats

The above data indicate that the increased phosphate loading to renal tubules promotes chemokine production, facilitating the migration of macrophage to renal tubules. Several lines of evidence indicate that mineral debris in tissues is cleared by macrophages and Kupffer cells^41,42^. It has been shown that macrophages from scavenger receptor-AI/AII (SR-A)-deficient mice have reduced clearance activity of CPP. Nonetheless, the precise mechanisms whereby macrophages efficiently recognize and remove mineral debris remain obscure. To obtain insights into the mechanisms, we finally compared global gene regulatory profiles in vivo in the kidney between DSH rats and DSH+2.5%SF rats by using an unbiased transcriptome analysis. We performed cDNA microarray analysis using pooled kidney RNAs isolated from three biological replicates in each group. Target genes were screened using *Z*-score criteria ≥ 2.0 or ≤−2.0 and a ratio ≥ 1.5 or ≤0.66, resulting in 543 upregulated and 532 downregulated targets in DSH+2.5% rats (compared with DSH rats). We then performed Kyoto Encyclopedia of Genes and Genomes (KEGG) pathway analysis to determine pathways in the kidneys that are affected. These analyses identified five pathways that were downregulated by SF with a *P* value of <0.05. Among the identified pathways, we excluded olfactory transduction because this pathway is likely to be picked up by chance due to abundance of the genes included in the pathway in rats.

The identified four pathways are shown in Table 2, which includes systemic lupus erythematosus, alcoholism, malaria, and African trypanosomiasis. A closer look at the identified gene list revealed that these genes can be classified into four groups: complement component genes, histone genes, genes encoding inflammatory mediators, and genes involved in erythropoiesis. The changes in histone genes (*Hist1h2ac*, *Hist2h2ab*, etc.) likely reflect the secondary tissue regeneration process in response to renal inflammation and damage. The changes in hemoglobin genes (*Hbb* and *Hbb-b1*) can be explained by the fact that SF contains Fe, which may affect erythropoiesis; however, we found that hemoglobin levels were not significantly different (Table 1).

**Table 2:** KEGG pathways downregulated in the kidney by SF.

Term	Count	Gene	P-value
Systemic lupus erythematosus	15	HIST1H2AC, RGD1564447, C4A, LOC498276, LOC102551184, LOC682330, LOC684762, C1QA, HIST2H2AB, HIST1H4B, LOC690131, HIST1H2AH, HIST1H2AK, HIST3H2A, HIST1H2AN	3.50E−07
Alcoholism	12	HIST1H2AC, HIST2H2AB, RGD1564447, HIST1H4B, HIST1H2AH, LOC690131, LOC102551184, HIST3H2A, HIST1H2AK, LOC682330, LOC684762, HIST1H2AN	8.10E−04
Malaria	6	ICAM1, CCL2, LOC100134871, LOC689064, HBB-B1, HBB	6.50E−03
African trypanosomiasis	5	ICAM1, LOC100134871, LOC689064, HBB-B1, HBB	6.90E−03

**Fig. 8:**
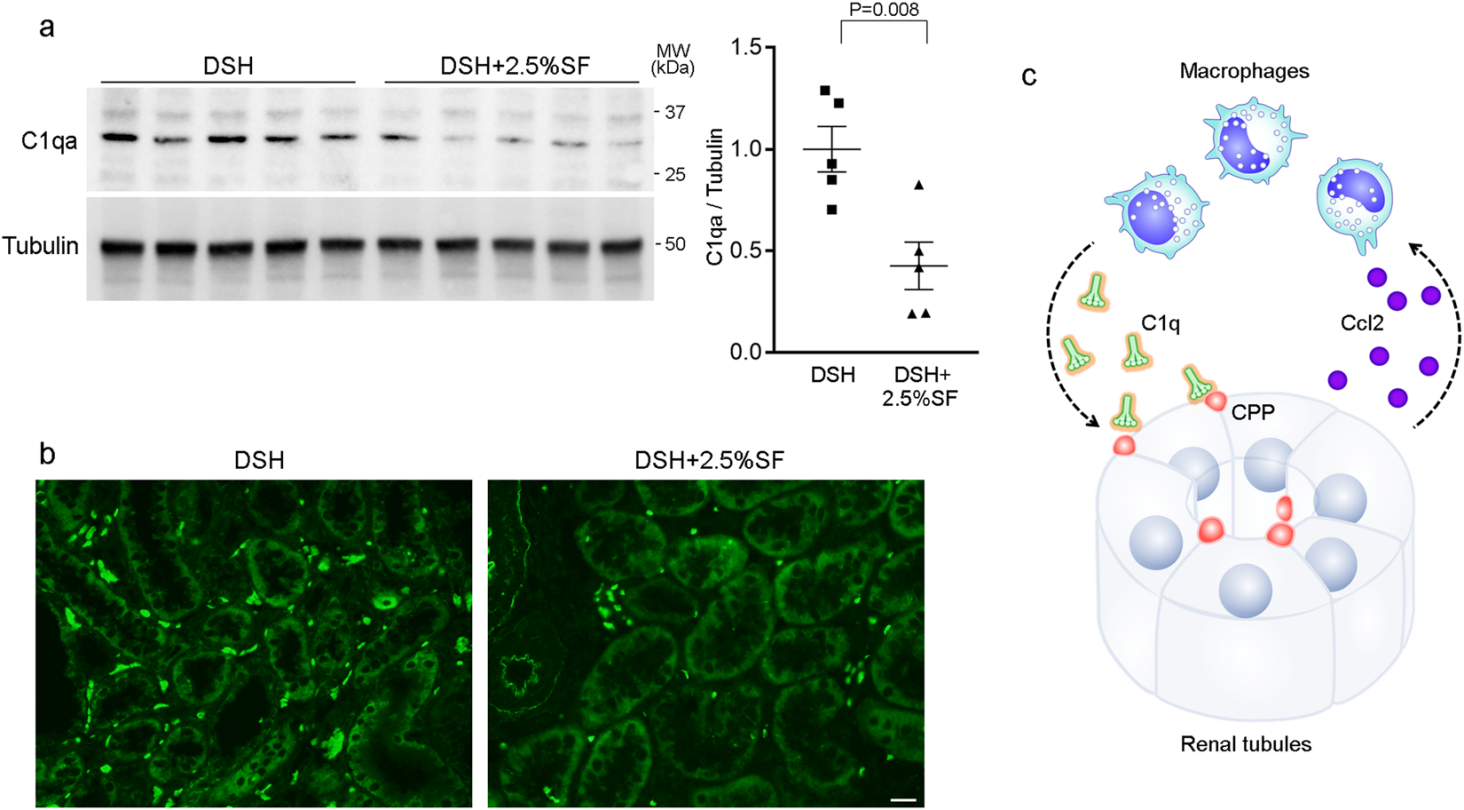
Possible involvement of complement in phosphate-mediated renal inflammation. **a** Renal levels of C1qa (upper panel) and tubulin (lower panel) were compared by western blotting between the DSH and DSH+2.5%SF groups. Dot plots show the results of quantitation. **b** Immunofluorescence study for C1qa in the indicated animals. Bars represent 20 μm. **c** A proposed model linking disturbed phosphate metabolism and tissue inflammation. Increased phosphate loading to renal tubules induces chemokine production including *Ccl2* in renal tubules likely through the formation of CaPi nanoparticles, thereby facilitating macrophage migration. Infiltrated macrophages not only contribute to the clearance of CaPi nanoparticle through complement-mediated opsonization but also cause renal inflammation. Data are expressed as mean ± SEM; *n* = 5 animals per group.

Among the identified genes, we noted with interest that genes encoding complement components were downregulated by SF. Besides classical roles in binding immune complex, accumulating data indicate that C1q recognizes and opsonizes debris such as oxLDL and amyloid-β protein^43–45^. Moreover, a recent study showed that C1q is a key component of CPPs formed in vivo^46^. Given the evidence, we evaluated tissue C1q levels in the kidney by western blot analysis. Consistent with the microarray data, we found that C1q protein levels were significantly lower in DSH+2.5%SF rats than in DSH rats (59% decrease; *P* = 0.008; Fig. 8a). IF study revealed that C1q was mainly observed in the tubulointerstitium, likely infiltrating macrophages, in DSH rats, which was attenuated in DSH+2.5%SF rats (Fig. 8b). These data demonstrate a potential role of the complement in the renal inflammation associated with the latent phosphate accumulation.

## Discussion

We here demonstrated that phosphate metabolism is dysregulated in Dahl/SS rats from an early stage, which contributes to the progression of kidney injury in this model. Although FGF-23 prevents the elevation of serum phosphate by increasing FE_P_, clinical studies suggested that its elevation in CKD is also associated with the decline in kidney function^2,4–6^. In the Mild to Moderate Kidney Disease study, FGF-23 predicted the progression of CKD independently of other factors, such as glomerular filtration rate (GFR), phosphate, and PTH^4^. Similarly, in the Chronic Renal Insufficiency Cohort study cohort, higher FGF-23 levels independently associated with greater risk of ESRD among participants with estimated GFR ≥ 30 ml/min/1.73 m^2 ^^5^. In participants of the community-based atherosclerosis Risk in Communities study, higher FGF-23 levels were shown to associate with increased risk of incident ESRD, independent of the baseline kidney function and other risk factors^6^. The current study is consistent with these reports and provides mechanistic insights into the association between FGF-23 and CKD progression. Because FGF-23 suppresses phosphate reabsorption in the kidney, its elevation results in the exposure of renal tubule cells to higher levels of phosphate, potentially promoting tissue inflammation.

We speculate phosphate itself or phosphate-containing factors such as CPPs in the urine contributed to the renal injury in our model. In the extracellular fluid, mineral-binding proteins such as fetuin-A adsorbs calcium phosphate nanocrystals to form CPPs, preventing growing into large crystals and precipitation^1,3^. Although these particles serve as a defense mechanism to prevent ectopic calcification, accumulating data suggest that CPPs can act as bioactive ligands that modify cellular function^29,40,42,46^. It has also been suggested that phosphaturia induced by FGF-23 exposes tubular cells to inappropriately high levels of phosphate, inducing tubular injury^2^. In Dahl/SS rats, serum phosphate levels were not elevated and were similar between the DSN and DSH groups. Consistently, plasma CPP levels were also similar among groups; however, latent phosphate accumulation in the DSH group was indicated by the marked increase in FE_P_ in this group. Furthermore, the degree of renal inflammation was well correlated with FE_P_ across the groups. Importantly, restricting intestinal phosphate absorption using SF suppressed FE_P_, attenuating renal inflammation and albuminuria (Figs. 1 and 2). Using proximal tubule cell line, moreover, we showed that phosphate loading to renal tubules directly induced *Ccl2* that was abolished by removing the calcium phosphate nanoparticles by centrifugation but not by the knockdown of NaPi2a transporter (Figs. 6 and 7). As reported previously, renal tubule cells are capable of producing Ccl2, and its elevation and binding to the cognitive receptor C-C chemokine receptor type 2 (CCR2) promote sustained inflammation and fibrosis through the cross-talk between damaged tubules and macrophages^47–49^. These data provide experimental evidence for the pathological importance of urinary phosphate and CPP in chronic kidney injury. Nonetheless, we did not directly determine CPP levels in the urine at the proximal tubules and distribution of CCR2 in the kidney, which requires future evaluation.

In CKD-mineral and bone disorder, several other factors have previously been implicated in the pathogenesis of cardiovascular and kidney injury that may potentially explain our observation. First, the elevation of phosphate and phosphate–calcium product could facilitate tissue damage through ectopic calcification. Indeed, the association of hyperphosphatemia with CKD progression is more pronounced in patients with high serum calcium levels, suggesting the role of ectopic calcification as the underlying mechanism^50^. Consistently, excessive phosphate intake induces nephropathy characterized by diffuse calcium–phosphate deposits in animal models^51^ and in humans^52,53^. However, we used a normal phosphorus diet throughout the experiment to prevent the occurrence of hyperphosphatemia and observed neither the increase in serum phosphate levels nor renal calcification in our model. Second, vitamin D might also play a role. Previous experimental studies suggested that the active form of vitamin D, 1,25(OH)_2_D_3_, can be protective against renal dysfunction through its anti-proliferative actions^54,55^. In our study, however, 1,25(OH)_2_D_3_ levels were not significantly different between the DSN and DSH groups. Third, direct toxic effects of FGF-23 have been reported in cardiovascular systems and in other cells outside the kidney^56,57^. Although we may not exclude the possibility that FGF-23 had direct injurious effects, our cell culture experiments clearly point to the importance of phosphate-containing factors in the pathogenesis.

In this study, we found that FGF-23 levels were suppressed in the DSH+2.5%SF group; however, HW/BW ratio was similarly increased in DSH and DSH+2.5%SF rats. The lack of effects on cardiac hypertrophy was confirmed by the evaluation of individual myofibril diameter. In contrast, macrophage infiltration was partially but significantly attenuated in DSH+2.5%SF rats (Fig. 5). The molecular mechanisms whereby SF attenuated cardiac inflammation is unclear but supports the importance of phosphate metabolism in cardio-renal interaction in Dahl/SS rats. As for cardiac remodeling, our data suggest that the elevation in blood pressure, but not FGF-23, plays a predominant role in cardiac hypertrophy in Dahl/SS rats, at least at an early stage. Given the evidence that the elevation of FGF-23 levels is associated with left ventricular hypertrophy^3^, however, the role of FGF-23 in cardiac remodeling at a later stage in this model needs further evaluation.

Another interesting observation in our study is that urinary phosphate excretion was highly induced by NaCl loading in Dahl/SS rats (Fig. 3b). This observation is in part explained by the significant increase in FGF-23 (Table 1) in response to modest reduction in glomerular filtration rate in the DSH group. However, given the magnitude of the effect (total urinary phosphate levels were higher in DSH than in DSN), it is likely that other mechanisms co-exist. Interestingly, a recent study reported that urinary phosphate excretion is profoundly increased in mice on a high-NaCl diet^58^, and neither FGF-23 nor PTH was altered by the diet in this acute-phase study. These data suggest that high salt intake can influence renal phosphate handling in both FGF-23-dependent and FGF-23-independent manners, although further studies are required to delineate the mechanisms. The current study demonstrates that the injurious effects of high salt intake are in part mediated by the altered phosphate metabolism in salt-sensitive hypertension.

As a potential mediator of macrophage infiltration associated with urinary phosphate, we found that C1q levels were increased in the DSH group compared with the DSH+2.5%SF group (Fig. 8). CPPs in the circulation have been shown to be cleared by macrophages through scavenger receptor-A^41^. Of note, a recent study showed that CPPs formed in vivo contain complement components including C1q^46^, suggesting the possibility that CPPs are opsonized with complement. The classical function of complement is to bind immune complexes, promoting the recognition and elimination of pathogens. Besides bacterial clearance, however, accumulating studies demonstrated that C1q can bind debris such as oxidized lipoproteins^43,45^, apoptotic cells^59^, and β-amyloid protein^44,60^, all of which are implicated in disease processes. In addition, aberrant C1q activity is associated with aging-related phenotypes^61^, a similar finding to that of abnormal phosphate metabolism^62^. Based on these data, we speculate that phosphate loading to renal tubules stimulates chemokine production such as Ccl2, promoting macrophage migration and complement induction (Fig. 8c). Although this process facilitates the clearance of phosphate-containing nanoparticles, it may result in renal inflammation, contributing to the progression of kidney disease.

There are several potential limitations in our study. Although we observed a profound increase in urinary phosphate levels in DSH rats, the mechanisms whereby high salt loading facilitates phosphaturia in salt-sensitive hypertension need further evaluation. We also acknowledge that microarray analysis was performed in limited samples. Despite these limitations, the current study clearly demonstrates that increased phosphaturia promotes inflammation and kidney injury from an early stage of salt-sensitive hypertension. Our data suggest that interventions against subclinical phosphate accumulation may improve the prognosis of hypertensive kidney disease.

## Methods

### Animal studies

Animal procedures were approved by the Teikyo University Ethics Committee for Animal Experiments (#15-027 and #20-007) and were conducted in accordance with the guidelines of Teikyo University. Male Dahl/SS hypertensive rats at 3 weeks of age were obtained from SLC (Tokyo, Japan). Dahl/SS rats received a standard chow (AIN-93G, which contained 0.5% of calcium, 0.3% of phosphorus, and 0.3% NaCl) served as a control (DSN). High-salt group received AIN-93G with 8% NaCl (DSH). The dose of high salt was determined based on our previous experiments^18^. A subgroup of DSH rats received SF (25 mg/g chow; DSH+2.5%SF) from 1 week after the beginning of DSH diet. We chose the dose because SF at 25 mg/g significantly reduced urinary phosphate without affecting serum phosphate levels, sodium intake, or weight gain in Dahl/SS rats (Table 1); serum phosphate levels were lower and weight gain was less at a higher dose. At the indicated periods, urine was collected for 24 h by using individual metabolic cages (Natsume KN-646, Japan).

Blood pressure was measured using volumetric pressure recording (CODA non-invasive Blood Pressure System; Kent Scientific, USA)^63^. This method has been validated to provide accurate blood pressure measurement and highly correlates with telemetry method^64^. To minimize the influence of diurnal variation, blood pressure was measured approximately at the same time in the afternoon. For each rat, we measured blood pressure values for 3 consecutive days to train the rats and calculated the mean ± SEM of ≥15 recordings.

At 4 weeks, animals were euthanized under anesthesia of inhaled isoflurane. After overnight fasting, blood samples were obtained by vena cava puncture. Kidneys and heart were removed, snap-frozen, and stored at −80 °C until use. Urinary albumin levels were measured by enzyme-linked immunosorbent assay (ELISA; SRL, Japan). Serum and urinary levels of electrolyte concentrations were measured by ion-selective electrode methods. Hemoglobin levels were measured by iSTAT blood gas analyzer (Abbott, USA). Serum intact PTH levels were measured by ELISA (Immutopics, USA). Serum intact FGF-23 levels were measured using the ELISA Kit (Kainos, Japan).

### Histology

For morphological evaluations, Methyl Carnoy’s solution-fixed, paraffin-embedded sections (1 μm) were stained with PAS reagent, and the degrees of glomerulosclerosis and tubulointerstitial injury were semiquantitatively analyzed in accordance with previous reports^19,65^. Glomerulosclerosis was defined as disappearance of cellular elements from the tuft, capillary loop collapse, and folding of the glomerular basement membrane with accumulation of amorphous material. The grades were 0, 0%; I, 1–25%; II, 26–50%; III, 51–75%; and IV, 76–100% of glomeruli involved. The glomerulosclerosis score was calculated as (1 × % grade I) + (2 × % grade II) + (3 × % grade III) + (4 × % grade IV). For each rat, 50 glomeruli were randomly analyzed. Tubulointerstitial injury was defined as tubular cast formation, tubular atrophy, or thickening of tubular basement membrane. The areas of the injured tubulointerstitium were calculated digitally using an image analysis program (ImageJ, 1.49v, National institutes of Health, USA), and the difference of quantified histological data between the DSH and DSH+2.5%SF groups was analyzed by unpaired *t* test (for tubulointerstitial injury area) or by Mann Whitney *U* test (for glomerulosclerosis score).

### Immunohistochemistry, IF staining, and quantification

Tissues were fixed in 4% paraformaldehyde for 4 h at 4 °C. Tissues were then incubated in 30% sucrose in phosphate-buffered saline overnight at 4 °C, mounted in OTC (Tissue-Tek), and were frozen until use. After blocking with Protein Block (DAKO, Denmark), cryosections (0.2 μm thick) were stained with the indicated primary antibodies and affinity-purified secondary antibodies-conjugated horseradish peroxidase (DAKO). Primary antibodies used included antibodies against CD68 (1:25, Serotec, UK), osteopontin (1:250, Developmental Studies Hybridoma Bank, USA), desmin (ready-to-use, DAKO)^18^, Ccl2 (1:100, Novus Biologicals), and C1qa (1:1000, Abcam, UK). For Quantification of CD68, at least 30 pictures at ×20 magnification for each rat were taken followed by digital quantification using the image analysis program (ImageJ, 1.49v, National institutes of Health, USA) and expressed as percentage of positively stained area. To quantify desmin in the glomeruli, at least 50 glomeruli at ×40 magnification were randomly selected from each rat. The ratio of pixels in the desmin-positive area per glomerulus was calculated using ImageJ.

IF study was performed on cryosections of the kidneys using antibodies against NHE3 (1:100, Abcam), NaPi2a (1:1000)^66^, NaPi2c (1:1000)^66^, CD68 (1:25, Serotec), and osteopontin (1:250, Developmental Studies Hybridoma Bank). Cryosections of the heart was stained with WGA (1:1000, Abcam, Cambridge, UK). Area of transversely cut muscle fibers was measured by ImageJ and GNU Image Manipulation Program 2.10.8 and averaged after determining in ten high-power fields.

### Western blotting

Kidneys were homogenized in buffer containing 10 mM Tris-HCl (pH 7.8), 1% Triton X, 150 mM NaCl, 1 mM EDTA, protease inhibitor cocktail (Roche), and phosphatase inhibitor (Sigma). The abundance of NaPi2a, NaPi2c, and NHE3 was analyzed in plasma membrane-enriched fraction that was purified from total kidneys using the Plasma Membrane Isolation Kit (Minute, Invent Biotechnologies, USA). Enrichment of plasma membrane proteins was validated in our previous studies^63,67^. Equal amounts of protein were mixed with Laemmli sample buffer, boiled for 5 min (or incubated at room temperature for membrane proteins), separated on polyacrylamide gel, and transferred to polyvinylidene difluoride membrane. The membrane was incubated with primary and peroxidase-conjugated secondary antibodies, followed by imaging using ECL reagents (Perkin Elmer, USA). Tubulin (1:2000, Sigma) and Ponceau S staining were used to ensure equal loading and transfer of different samples. We used monoclonal antibody against Klotho (1:500, KM2119, Trans Genic, Japan)^29^ and antibodies against C1qa (1:5000, ab189922, Abcam, UK), NaPi2a (1:1000, NPT27A, alpha-Diagnostics, USA), NaPi2c (1:7500)^66^, and NHE3 (1:1000, 3H3, Millipore). Original images of the blots are shown in Supplementary Fig. 4.

### Measurement of plasma CPP

Plasma levels of CPPs were measured in accordance with Miura et al.^30^. Plasma (5 μl) was added to 45 μl of Dulbecco’s modified Eagle’s medium (DMEM) containing 100 mM HEPES (pH 8.0) supplemented with 0.5 μM OsteoSense 680EX (PerkinElmer) and incubated at 25 °C for 60 min. The mixture was then applied to a gel-filtration spin column (Bio-rad, molecular weight cut-off 40 kDa) and centrifuged at 1000 × *g* for 2 min. The flow-through fraction was diluted with the same volume of 2% sodium dodecyl sulfate and 100 mM EDTA. The fluorescence intensity of OsteoSense was quantified using an infrared fluorescence scanner.

### Cell culture and phosphate loading

NRK-52E cells (obtained from American Type Culture Collection, VA) were cultured in DMEM (Thermo Fisher Scientific, USA) supplemented with 10% heat-inactivated fetal bovine serum (FBS). When indicated, Pi concentration in the media containing FBS was increased using 0.5 mol/l sodium phosphate, pH 7.4 (a mixture of NaH_2_PO_4_ and Na_2_HPO_4_). Basal DMEM with 10% FBS contains ~1.0 mmol/l Pi. Unless otherwise stated in the results, “high-Pi” medium refers to a medium with an additional 2 mmol/l Pi (~3 mmol/l final). The dose was based on previous studies that test the effects of phosphate loading in cell culture^40,68,69^ and also based on our observation that urinary phosphate levels were several times higher in DSH than in DSN.

### RNAi studies

NRK-52E cells were plated in 6-well plates (100,000 per well). After 24 h, *Slc34a1* RNAi encoding NaPi2a (which is a major form of renal phosphate transporter in rodents)^39^ or control RNAi (both from Dharmacon, On-Target plus, USA) was introduced by using TransIT-X2 reagent. Specificity of siRNA is validated by vendor and is further confirmed in the present study. siRNA (5 μl of 20 μM concentration) and TransIT-X2 reagent (10 μl) were mixed in Opti-MEM (250 μl). After 30 min of incubation, the mixture was added to 2.5 ml of the cell culture medium. After another 24 h, cells were then incubated in DMEM containing different concentrations of Pi as indicated.

### RNA extraction and quantitative RT-PCR

RNA was extracted using the RNeasy Kit (Qiagen, USA). The cDNA was synthesized from 1 μg of total RNA using the High Capacity cDNA Reverse Transcription Kit (Thermo Fisher Scientific). TaqMan gene expression assays (Thermo Fisher Scientific) were used for the quantitative RT-PCR analysis. Gene expression was quantitatively analyzed by real-time RT-PCR using 7500 Fast Real-time PCR System (Applied Biosystems). Relative quantification was accomplished with measurement of the threshold cycle and use of the standard curve. The expression levels of the target genes were normalized to those of *Actb*.

### Microarray analysis

Total RNA was isolated from the whole kidney as described above. cDNA microarray analysis was performed at Cell Innovator (Fukuoka, Japan). The cRNA was amplified, labeled with total RNA using the GeneChip™ WT PLUS Reagent Kit (Thermo Fisher), and hybridized to Thermo Fisher Scientific Clariom™ D Assay according to the manufacturer’s instructions. All hybridized microarrays were scanned by an Affymetrix scanner. Relative hybridization intensities and background hybridization values were calculated using Thermo Fisher Expression Console™. Target genes were selected with *Z*-score criteria ≥ 2.0 or ≤−2.0 and a ratio ≥ 1.5 or ≤0.66, followed by KEGG pathway analysis.

### Statistics and reproducibility

The data are summarized as mean ± SEM. GraphPad Prism software, version 7.05 (GraphPad Software Inc.) was used for statistical analyses. Unpaired *t* test was used for comparisons between two groups. Non-parametric data were analyzed by Mann–Whitney *U* test. For multiple comparisons, statistical analysis was performed by analysis of variance followed by Tukey post hoc tests. Correlation between parameters was analyzed by Pearson’s correlation test. A *P* value < 0.05 (two sided) was considered statistically significant.

### Reporting summary

Further information on research design is available in the Nature Research Reporting Summary linked to this article.

## Supplementary information

Supplementary Information

Description of Additional Supplementary Files

Supplementary Data 1

Reporting summary

## Data Availability

Raw images of the western blots are provided in Supplementary Fig. 4. Source data underlying the graphs are provided in Supplementary Data 1. Raw microarray data are available at the NCBI Gene Expression Omnibus (GEO) under accession GSE143440. Other relevant data are available from the corresponding author upon request.
