# Supplementary material for: Urinary phosphate-containing nanoparticle contributes to inflammation and kidney injury in a salt-sensitive hypertension rat model
Source: Commun Biol. 2020 Oct 15;3:575. doi: 10.1038/s42003-020-01298-1 (PMC7562875; doi:10.1038/s42003-020-01298-1)

Supplementary Figures for:

Urinary phosphate-containing nanoparticle contributes to inflammation and kidney injury in a salt-sensitive hypertension rat model

Qin Wang[a,b], Kenichi Ishizawa[a], Jinping Li[a,c], Wataru Fujii[a], Yoshikazu Nemoto[a], Osamu Yamazaki[a], Yoshifuru Tamura[a], Yutaka Miura[d], Xuedan Nie[e], Ryo Abe[f], Hiroko Segawa[g], Makoto Kuro-O[d], Shigeru Shibata[a]

[a]Division of Nephrology, Department of Internal Medicine, Teikyo University School of Medicine, Tokyo 173-8605, Japan. [b]Department of Nephrology, the Second Affiliated Hospital of Harbin Medical University, Harbin 150081, China. [c]Department of Nephrology, Tianjin First Central Hospital, Tianjin 300000, China. [d]Division of Anti-aging Medicine, Center for Molecular Medicine, Jichi Medical University, Tochigi 329-0498, Japan. [e]Department of Neurology, the Second Affiliated Hospital of Harbin Medical University, Harbin 150081, China. [f]Strategic Innovation and Research Center, Teikyo University, Tokyo 173-8605, Japan. [g]Department of Applied Nutrition, Institute of Biomedical Sciences, Tokushima University Graduate School, Tokushima 770-8503, Japan.

Supplementary Figure 1.

Representative micrographs of immunostaining for desmin, a marker for glomerular podocyte injury. Dot plots show the results of quantitative evaluation of desmin staining in the glomeruli. Data are expressed as mean ± SEM.

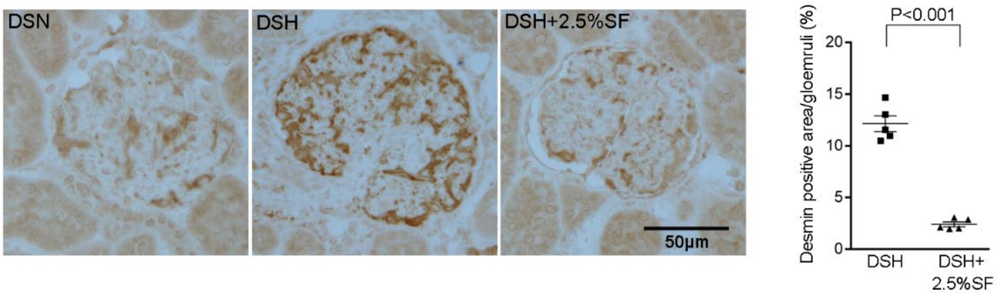

Supplementary Figure 2.

Calciprotein particle (CPP) levels in DSH rats. (a) Plasma calciprotein particle (CPP) levels in the indicated groups. (b, c) Correlation between CPP levels and serum phosphate (b) or Ca x Pi product (c). Data are expressed as mean ± SEM. NS, not significant.

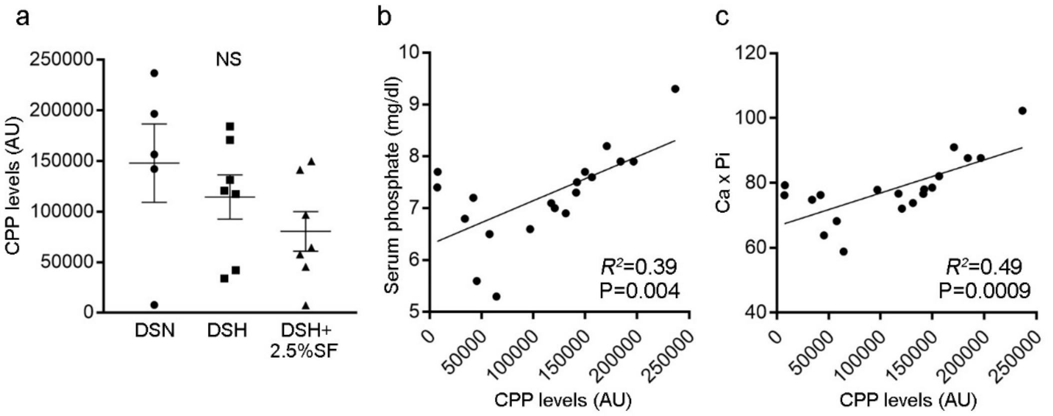

Supplementary Figure 3.

Effects of phosphate loading on *Opn* expression in proximal tubule cells. NRK-52E cells were incubated with high inorganic phosphate (Pi) for 1h, 6h, and 24h. Expression levels were normalized to those of *Actb*. Ctrl, control; Data are expressed as mean ± SEM.

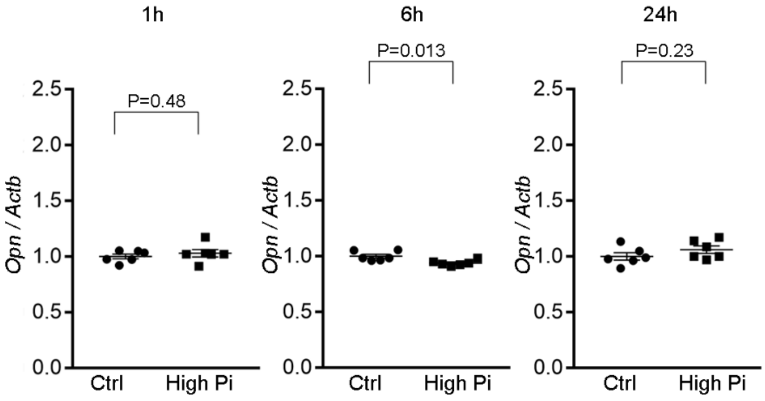

Supplementary Figure 4.

Original images of the Western blot analysis used in Fig.4, Fig.7, and Fig.8.

Klotho (Fig.4a)

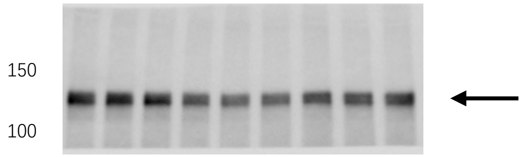

Tubulin (Fig.4a)

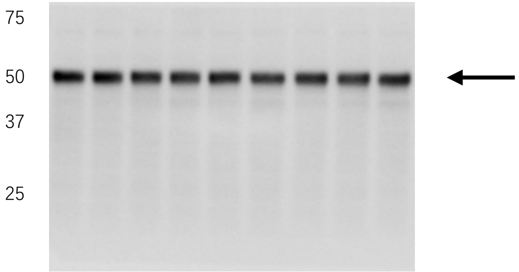

NaPi2a (Fig.4b)

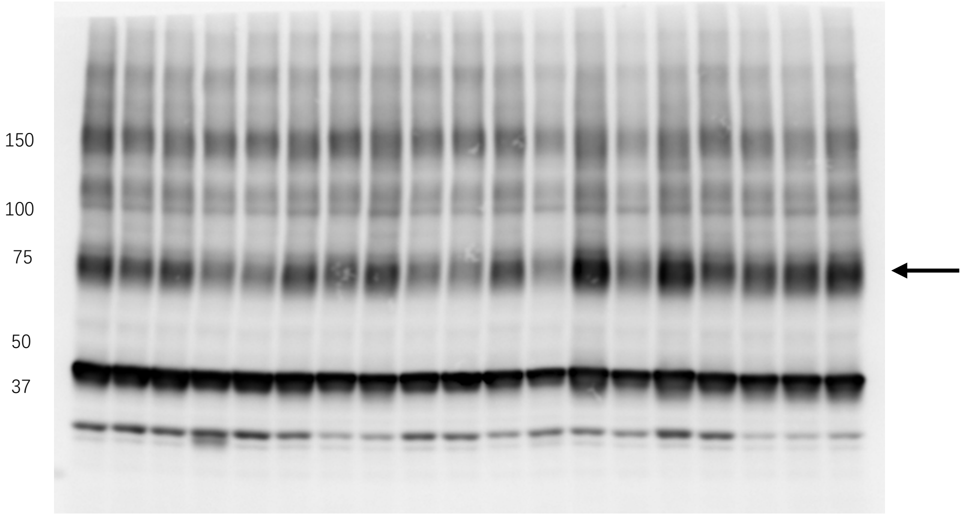

NaPi2c (Fig.4b)

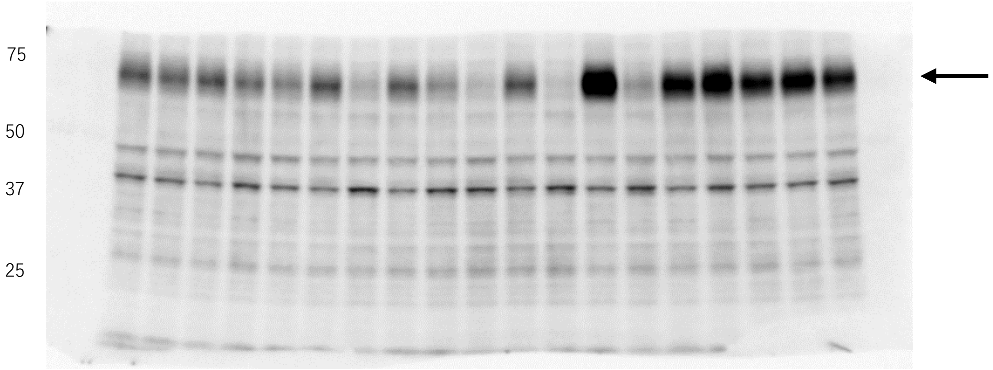

NHE3 (Fig.4b)

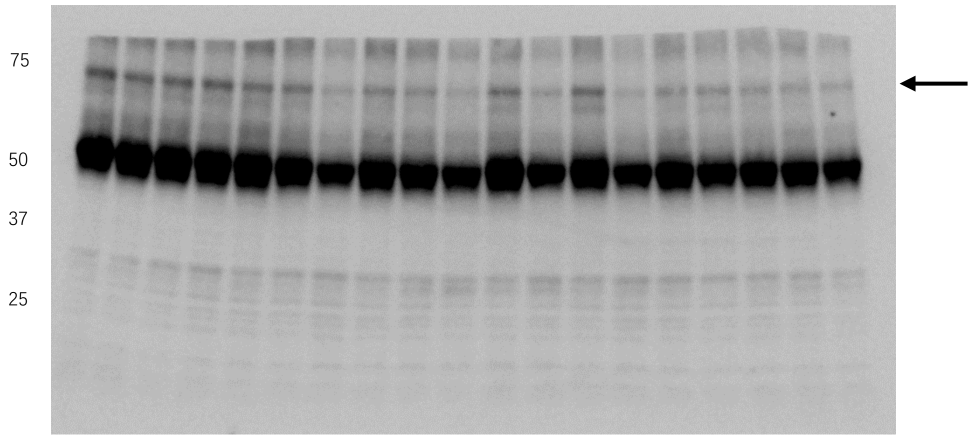

NaPi2a (Fig.7b)

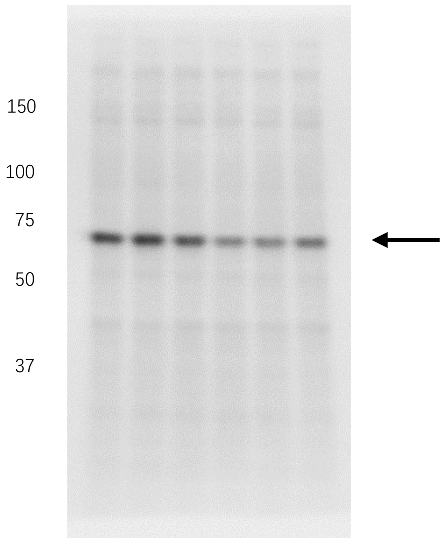

NaPi2c (Fig.7b)

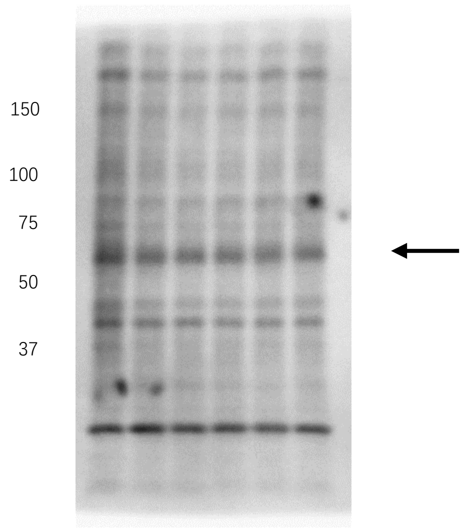

Tubulin (Fig.7b)

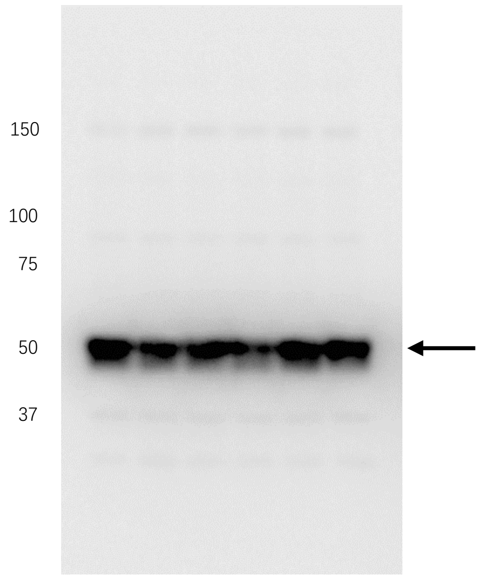

C1qa (Fig. 8a)

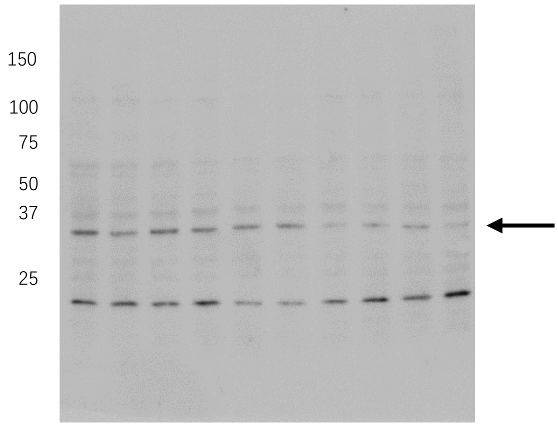

Tubulin (Fig. 8a)

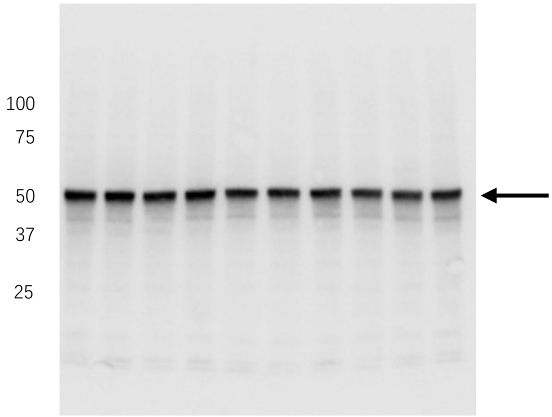

Supplement: Supplementary file 1 — Supplementary Information [file 42003_2020_1298_MOESM1_ESM.pdf]
