# Supplementary material for: Urinary phosphate-containing nanoparticle contributes to inflammation and kidney injury in a salt-sensitive hypertension rat model
Source: Commun Biol. 2020 Oct 15;3:575. doi: 10.1038/s42003-020-01298-1 (PMC7562875; doi:10.1038/s42003-020-01298-1)
Supplement: Supplementary file 2 — Description of Additional Supplementary Files [file 42003_2020_1298_MOESM2_ESM.pdf]

Description of Additional Supplementary Files

File Name: Supplementary Data 1
Description: This file includes all source data underlying the graphs and charts presented in the main figures
