# Supplementary material for: Urinary phosphate-containing nanoparticle contributes to inflammation and kidney injury in a salt-sensitive hypertension rat model
Source: Commun Biol. 2020 Oct 15;3:575. doi: 10.1038/s42003-020-01298-1 (PMC7562875; doi:10.1038/s42003-020-01298-1)
Supplement: Supplementary file 4 — Reporting summary [file 42003_2020_1298_MOESM4_ESM.pdf]

nature research

Corresponding author(s): Shigeru Shibata

Last updated by author(s): Aug 24, 2020

Reporting Summary

Nature Research wishes to improve the reproducibility of the work that we publish. This form provides structure for consistency and transparency in reporting. For further information on Nature Research policies, see our Editorial Policies and the Editorial Policy Checklist.

Statistics

For all statistical analyses, confirm that the following items are present in the figure legend, table legend, main text, or Methods section.

n/a	Confirmed	
☐	☒	The exact sample size (n) for each experimental group/condition, given as a discrete number and unit of measurement
☐	☒	A statement on whether measurements were taken from distinct samples or whether the same sample was measured repeatedly
☐	☒	The statistical test(s) used AND whether they are one- or two-sided<br>*Only common tests should be described solely by name; describe more complex techniques in the Methods section.*
☒	☐	A description of all covariates tested
☒	☐	A description of any assumptions or corrections, such as tests of normality and adjustment for multiple comparisons
☐	☒	A full description of the statistical parameters including central tendency (e.g. means) or other basic estimates (e.g. regression coefficient) AND variation (e.g. standard deviation) or associated estimates of uncertainty (e.g. confidence intervals)
☒	☐	For null hypothesis testing, the test statistic (e.g. F, t, r) with confidence intervals, effect sizes, degrees of freedom and P value noted<br>*Give P values as exact values whenever suitable.*
☒	☐	For Bayesian analysis, information on the choice of priors and Markov chain Monte Carlo settings
☒	☐	For hierarchical and complex designs, identification of the appropriate level for tests and full reporting of outcomes
☒	☐	Estimates of effect sizes (e.g. Cohen's d, Pearson's r), indicating how they were calculated

Our web collection on statistics for biologists contains articles on many of the points above.

Software and code

Policy information about availability of computer code

Data collection	No software was used.
Data analysis	The data were analyzed with GraphPad Prism (vresion 7.0.5), ImageJ, GIMP (version 2.10.8), Thermo Fisher Expression Console, and DAVID 6.8.

For manuscripts utilizing custom algorithms or software that are central to the research but not yet described in published literature, software must be made available to editors and reviewers. We strongly encourage code deposition in a community repository (e.g. GitHub). See the Nature Research guidelines for submitting code & software for further information.

Data

Policy information about availability of data

All manuscripts must include a data availability statement. This statement should provide the following information, where applicable:

- Accession codes, unique identifiers, or web links for publicly available datasets
- A list of figures that have associated raw data
- A description of any restrictions on data availability

Raw data of the Western blots are provided in Supplementary Fig.4. Source data underlying the graphs are provided in Supplementary Data 1. Raw data of microarrays are available at GEO (GSE143440). Other relevant data are available from the corresponding author upon request.

Field-specific reporting

Please select the one below that is the best fit for your research. If you are not sure, read the appropriate sections before making your selection.

☒ Life sciences ☐ Behavioural & social sciences ☐ Ecological, evolutionary & environmental sciences

For a reference copy of the document with all sections, see nature.com/documents/nr-reporting-summary-flat.pdf

Life sciences study design

All studies must disclose on these points even when the disclosure is negative.

Sample size	Sample sizes were determined based on our previous studies.
Data exclusions	No data were excluded.
Replication	The experimental findings were reliably reproduced.
Randomization	Rats were randomly assigned to three groups.
Blinding	No blinding was performed.

Reporting for specific materials, systems and methods

We require information from authors about some types of materials, experimental systems and methods used in many studies. Here, indicate whether each material, system or method listed is relevant to your study. If you are not sure if a list item applies to your research, read the appropriate section before selecting a response.

Materials & experimental systems

n/a	Involved in the study
☐	☒ Antibodies
☐	☒ Eukaryotic cell lines
☒	☐ Palaeontology and archaeology
☐	☒ Animals and other organisms
☒	☐ Human research participants
☒	☐ Clinical data
☒	☐ Dual use research of concern

Methods

n/a	Involved in the study
☒	☐ ChIP-seq
☒	☐ Flow cytometry
☒	☐ MRI-based neuroimaging

Antibodies

Antibodies used	CD68 (FA-11, Serotec); osteopontin (MPIIIB10, Developmental Studies Hybridoma Bank); desmin (D33, DAKO); Ccl2 (NBP1-07025, Novus Biologicals); NHE3 (Ab95299, Abcam); wheat germ agglutinin (Abcam, Cambridge, UK); NaPi2a (NPT27A, alpha-Diagnostics, USA); Tubulin (T9026, Sigma); Klotho (KM2119, Trans Genic); C1qa (ab189922, Abcam, UK); NHE3 (3H3, Millipore); NaPi2a (provided by Dr. Segawa); NaPi2c (provided by Dr. Segawa).
Validation	All antibodies were reasonably validated in previous studies or by vendors.

Eukaryotic cell lines

Policy information about cell lines

Cell line source(s)	NRK-52E cells were obtained from American Type Culture Collection, VA.
Authentication	Cell line was authenticated by the manufacturer.
Mycoplasma contamination	The cell line was tested negative for mycoplasma.
Commonly misidentified lines (See ICLAC register)	No commonly misidentified cell line was used.

Animals and other organisms

Policy information about studies involving animals; ARRIVE guidelines recommended for reporting animal research

Laboratory animals	Male Dahl salt-sensitive hypertensive rats (Dahl/SS) at 3 weeks of age were obtained from SLC (Tokyo, Japan).
Wild animals	The study did not involve wild animals.
Field-collected samples	The study did not involve samples collected from the field.
Ethics oversight	Animal procedures were approved by the Teikyo University Ethics Committee for Animal Experiments and were conducted in accordance with the guidelines of Teikyo University.

Note that full information on the approval of the study protocol must also be provided in the manuscript.
